# Small Proline-Rich Protein 3 Regulates IL-33/ILC2 Axis to Promote Allergic Airway Inflammation

**DOI:** 10.3389/fimmu.2021.758829

**Published:** 2022-01-20

**Authors:** Guiping Zhu, Hui Cai, Ling Ye, Yuqing Mo, Mengchan Zhu, Yingying Zeng, Xixi Song, Chengyu Yang, Xin Gao, Jian Wang, Meiling Jin

**Affiliations:** Department of Pulmonary and Critical Care Medicine, Zhongshan Hospital, Fudan University, Shanghai, China

**Keywords:** asthma, SPRR3, airway inflammation, group 2 innate lymphoid cell, PI3K/AKT/NF-κB pathway

## Abstract

Small proline-rich proteins (SPRRs), components of cornified cell envelope precursors, have recently been found to participate in airway diseases. However, their role in allergic airway inflammatory conditions remains unknown. Here, we explored the expression of SPRR3 in house dust mite (HDM)-sensitized/challenged mice and attempted to elucidate the regulatory role of SPRR3 in allergic airway inflammation. SPRR3 was identified *via* bioinformatics analysis of Gene Expression Omnibus (GEO) databases and further confirmed to be upregulated in the lungs of asthmatic mice. Knockdown of SPRR3 *via* the intratracheal route significantly inhibited eosinophils in bronchoalveolar lavage fluid (BALF) and suppressed the expressions of type 2 cytokines (IL-4, IL-5, and IL-13) in BALF and lung tissues. Further, SPRR3 knockdown reduced the expression of IL-33 and further attenuated the activation of the PI3K/AKT/NF-κB signaling pathway in the recruitment of group 2 innate lymphoid cells (ILC2s) to inhibit allergic airway inflammation. *In vitro*, SPRR3 siRNA could alleviate HDM-induced inflammatory responses in BEAS-2B cells. This study reveals the regulatory role of SPRR3 in allergic airway inflammation, identifying this protein as a potential novel therapeutic target for asthma.

## Introduction

Asthma, a chronic airway disease, is characterized by airway inflammation and airway hyperresponsiveness. It is a heterogeneous disease with complex endotypes, among which type 2 high inflammation accounts for the majority of asthma cases (1, 2). During the allergen challenge, alarmins such as IL-25, IL-33, and thymic stromal lymphopoietin (TSLP) are released from airway epithelial cells, leading to the activation of group 2 innate lymphoid cells (ILC2s) and Th2 cells and overproduction of IL-4, IL-5, and IL-13, consequently promoting Th2 inflammatory responses. Airway type 2 immune environments are characterized by eosinophil recruitment and infiltration, antigen-specific immunoglobulin E (IgE) production, goblet cell hyperplasia, and airway hyperresponsiveness (3–5).

Airway epithelial cells are the first line of defense against inhaled allergens and environmental exposure in asthma (6). Many of the identified susceptibility genes for asthma are expressed in the airway epithelium, supporting the notion that airway epithelial cells are critical for the development of the disease (7). The epithelial cell-derived alarmins, including IL-25, IL-33, and TSLP, play pivotal roles in connecting adaptive immunity and innate immunity (8). However, the mechanism by which IL-25, IL-33, and TSLP are regulated in bronchial epithelial cells remains unknown. Identifying the epithelial-derived mediator that regulates the expression of these cytokines is of great significance for understanding the molecular mechanism by which asthma is initiated.

Small proline-rich proteins (SPRRs), which are components of cornified cell envelope precursors, are expressed in the normal epithelium from several organs, including the esophagus, mouth, larynx, and lung (9, 10). Current studies of SPRRs mainly focus on tumors and atopic dermatitis. SPRRs may be related to the proliferation and migration of tumor cells and skin barrier dysfunction in atopic dermatitis (11, 12). Recently, the role of SPRRs in chronic inflammation has also been reported. Both SPRR1 and SPRR2A participated in mucosal inflammation in chronic rhinosinusitis, potentially leading to epithelial barrier dysfunction (13). Zimmermann *et al.* found that SPRR2A and SPRR2B were upregulated in an IL-13-dependent manner in an allergen-induced asthma model (14). Furthermore, SPRR2B polymorphisms may serve as an important predictive marker for asthmatic children with eczema (15). Other evidence indicated that SPRR3 was upregulated in the airway epithelium of smokers, which could contribute to the development of chronic obstructive pulmonary disease (COPD) (16). These findings suggest that SPRRs have a substantial association with the occurrence of chronic airway inflammatory diseases including asthma, although the causal relationships between SPRRs and airway epithelial cells (especially epithelial-derived cytokines) remain unclear. Thus, it is necessary to elucidate the functions of these proteins in airway epithelial inflammation.

In this study, we employed bioinformatics analysis using the Gene Expression Omnibus (GEO) database to evaluate the differentially expressed genes in human bronchial epithelial cells between patients with asthma and healthy subjects and confirmed that SPRR3 was significantly increased in allergic airway inflammation induced by house dust mite (HDM). To further explore the role of SPRR3 in eosinophilic airway inflammation, we performed a knockdown of SPRR3 expression in the airway epithelium and examined downstream mechanisms. The findings may identify a new target for the treatment of asthma.

## Materials and Methods

### Screening of Differentially Expressed Genes

GSE64913 and GSE37693 datasets were selected from the GEO database (https://www.ncbi.nlm.nih.gov/geo/). The GSE64913 dataset comprised transcriptomic analyses of epithelial brushings obtained from healthy controls and asthma patients, while the GSE37693 dataset was derived from primary culture airway epithelial cells treated with or without IL-13. GEO2R (http://www.ncbi.nlm.nih.gov/geo/geo2r/), an R-based web application for analysis of GEO data, was applied to compare and screen differentially expressed genes (DEGs) between asthma patients and healthy individuals. *p*-Value <0.05 and |Log fold change (FC)| ≥ 1 was set as the threshold for identifying DEGs. A Venn diagram was utilized to analyze the overlap of DEGs between asthmatic bronchial epithelial cells and IL-13-treated airway epithelial cells. The common differential genes were defined as candidate DEGs, and the Hiplot website was used to produce a heatmap of them.

### Experimental Animals

Female C57BL/6 mice (6–8 weeks) weighing 18–22 g were purchased from JSJ Laboratory Animals (Shanghai, China). Mice were acclimatized for a week and maintained in specific pathogen-free conditions at the Animal Center of Zhongshan Hospital, Fudan University. All experiments were approved by the Animal Care and Use Committee of Zhongshan Hospital and performed in accordance with institutional guidelines.

### Animal Experimental Protocols

To establish the HDM-induced allergic airway inflammation model, mice were anesthetized with isoflurane before intranasal aspiration of 40 μl of lyophilized HDM extract solution (0.25 μg/μl; Greer Laboratories) on days 0, 1, and 2 and then days 8 to 12. On days 8, 10, and 12, mice were transfected intranasally with 20 μg of siRNA dissolved with transfection reagent (Polyplus-transfection, France) 2 h before the HDM challenge. The control group was sensitized and challenged with phosphate-buffered saline (PBS) alone. All mice were anesthetized with avertin (25 mg/kg, Sigma-Aldrich, St. Louis, MO, USA) and sacrificed for analysis 48 h after the last HDM challenge. The sequence of SPRR3 siRNA was as follows: forward 5′-GAGCCAGAUUAUACUACAATT-3′, reverse 5′-UUGUAGUAUAAUCUGGCUCTT-3′.

### Differential Counting of Cells in Bronchoalveolar Lavage Fluid

Two days after the last HDM challenge, bronchoalveolar lavage fluid (BALF) was collected by gentle injection of 1 ml of PBS into the trachea and lungs through a 22-inch intravenous catheter. The supernatant of BALF was collected by centrifugation at 300 g for 10 min at 4°C and then stored at −80°C for further analyses. Total numbers of cells in BALF were counted using CellDrop^®^ (DeNovix, Wilmington, DE, USA). Flow cytometry was used to classify BALF cells, with eosinophils marked as CD45^+^Ly6G^−^CD11c^−^SiglecF^+^ and neutrophils marked as CD45^+^Ly6G^+^. The results for differential counting of BALF cells are shown as the number of cells per milliliter of BALF.

### Histopathological Evaluation of Lung Tissue

The left upper lung lobe was fixed in 4% paraformaldehyde and embedded in paraffin. Sections were stained with H&E and periodic acid-Schiff (PAS) to assess airway inflammation and mucus secretion. To grade the extent of lung inflammation and goblet cell hyperplasia, a semiquantitative scoring system was used as previously described (17, 18). All images were examined in a random blind fashion by 2 independent investigators.

### Immunohistochemistry Staining

Lung tissues were fixed with 4% paraformaldehyde and subsequently embedded in paraffin for immunohistochemistry staining using a rabbit anti-SPRR3 (mouse) antibody (1:1,000; Servicebio) to assess the expression of SPRR3.

### Flow Cytometry of Lung ILC2s and CD4^+^ T cells

Flow cytometry of lung ILC2s was performed as in our previous study (19). Lung tissues were prepared as single-cell suspensions. After treatment with red blood cell (RBC) lysis solution and incubation with CD16/32 monoclonal antibody (MultiSciences, China) for 10 min to block Fc receptors, cells were stained with a mixture of APC-eFluor 780-conjugated anti-CD45 (30-F11, eBioscience), PerCP-cy5.5-conjugated anti-lineage markers (CD3e, CD11b, CD45R/B220, Ly-76, Ly-6G, and Ly-6C) (BD Pharmingen), fluorescein isothiocyanate (FITC)-conjugated anti-Thy1.2 (53–2.1, eBioscience), and APC-conjugated anti-ST2 (DIH9, BioLegend) for 30 min. ILC2s were identified as CD45^+^Lin^−^Thy1.2^+^ST2^+^ as previously described (19–21). CD4^+^ T cells were stained with a mixture of Percp-cy5.5-conjugated anti-CD3e (BioLegend), BV510-conjugated anti-CD4 (BD Bioscience), and APC-cy7-conjugated anti-CD45 (BioLegend) for 30 min on ice. The samples were acquired by Aria II flow cytometers (BD Biosciences, San Jose, CA, USA) and analyzed with FlowJo software (version10.0, Tree Star, Inc., Ashland, OR, USA).

### Cytokine Measurement

Levels of type 2 cytokines (IL-4, IL-5, and IL-13) and IL-33 in BALF supernatants were analyzed by ELISA (MultiSciences, China) following the manufacturer’s instructions. SPRR3 expression in BALF was analyzed by ELISA (Nova Lifetech, Hong Kong).

### RNA Isolation and Quantitative PCR Analysis

The total RNA was extracted from lung tissue homogenates using TRIzol Reagent and real-time PCR was performed using a SYBR PCR Kit (Yeasen Biotech, Shanghai, China) according to the manufacturer’s instructions. The expression of GAPDH was used as an internal reference. The primer sequences of all genes for PCR were as follows: mouse GAPDH forward 5′-GGGTGTGAACCACGAGAAAT-3′, reverse 5′-CCTTCCACAATGCCAAAGTT-3′; mouse IL-4 forward 5′-TACCAGGAGCCATATCCACGGATG-3′, reverse 5′-TGTGGTGTTCTTCGTTGCTGTGAG-3′; mouse IL-5 forward 5′-GGCCACTGCCATGGAGATTCC-3′, reverse 5′-AGCCTCATCGTCTCATTGCTTGTC-3′; mouse IL-13 forward 5′-CGGCAGCATGGTATGGAGTGTG-3′, reverse 5′-GGAGGCTGGAGACCGTAGTGG-3′; mouse IL-33 forward 5′-TCCAACTCCAAGATTTCCCCG-3′, reverse 5′-CATGCAGTAGACATGGCAGAA′; human GAPDH forward 5′-CCACCCATGGCAAATTCCATGGCA-3′, reverse 5′-TCTACACGGCAGGTCAGGTCCACC-3′; human TSLP forward 5′-CGGCCACATTGCCTTACTGA-3′, reverse 5′-TAGCCTGGGCACCAGATAGC-3′; human IL-25 forward 5′-CTGAGGAGCTGCTGAGGTGGAG-3′, reverse 5′-GCCGGTTCAAGTCTCTGTCCAAC-3′; human IL-33 forward 5′-CTGGTACTCGCTGCCTGTCAAC-3′, reverse 5′-ACCATCAACACCGTCACCTGATTC-3′; human SPRR3 forward 5'-ATGAGTTCTTACCAGCAGAAGC-3',reverse 5'-GTTCAGGGACCTTGGTGTAGC-3'.

### Western Blotting

Mouse lungs were lysed in radioimmunoprecipitation assay (RIPA) buffer (Beyotime, China) containing phenylmethane-sulfonyl fluoride (PMSF; Beyotime, China) and phosphatase inhibitor (Roche, Germany). After incubation for 30 min on ice, lysates were centrifuged at 12,000 rpm for 15 min to remove insoluble material. Expression of different proteins in mouse lungs was measured by Western blotting on 10% sodium dodecyl sulfate–polyacrylamide gel electrophoresis (SDS-PAGE) gels. After transferring to a polyvinylidene difluoride (PVDF) membrane and blocking, proteins were incubated with primary antibodies against SPRR3 (BioWorld, USA) and phospho-AKT, AKT, phospho-NF-κB-P65, NF-κB-P65, and β-actin (CST, Danvers, MA, USA). Antibodies were detected using horseradish peroxidase-conjugated anti-rabbit or anti-mouse IgG (Beyotime, China) followed by ECL Western blotting detection reagent (Beyotime Biotech, China). Densitometry was performed using ImageJ (National Institutes of Health, USA), and the relative expressions of p-AKT and p-NF-κB-P65 were normalized by comparison with total AKT and total NF-κB-P65, respectively.

### Cell Culture and Treatment

BEAS-2B cells were purchased from the Chinese Academy of Sciences (Shanghai, China) and cultured in Dulbecco’s modified Eagle medium (DMEM)/high glucose (HyClone, Logan, UT, USA), supplemented with 10% fetal bovine serum (Gibco, Waltham, MA, USA) and 1% penicillin/streptomycin (Gibco) at 37°C in a culture chamber containing 5% CO_2_. When the cell confluence reached 50% in 12-well plates, a mixture of 2 μl of SPRR3 siRNA (20 μmol/L) or NC siRNA diluted in 100 μl of Opti-MEM (Gibco) and 2 μl of Lipofectamine 2000 (Invitrogen) in 100 μl of Opti-MEM was added to the basal medium of each well. Six hours later, the medium was substituted with complete DMEM. Two hours before the end of the culture, cells were treated with HDM (40 μg/ml) and then harvested for quantitative PCR. The sequence of SPRR3 siRNA was as follows: forward 5′-GACCAAGGCUUCAAGUTT-3′, reverse 5′-ACUUGAUGAAGCCUUGGUCTT-3′.

### Statistical Analysis

Data were presented as mean ± standard error of the mean (SEM). Statistical analyses were performed *via* parametric Student’s t-test or one-way ANOVA with Tukey’s multiple comparison test using GraphPad Prism (version 8). In all cases, *p*-values of 0.05 and below were considered to indicate significance.

## Results

### Screening and Confirmation of Differentially Expressed Genes

**Figure 1:**
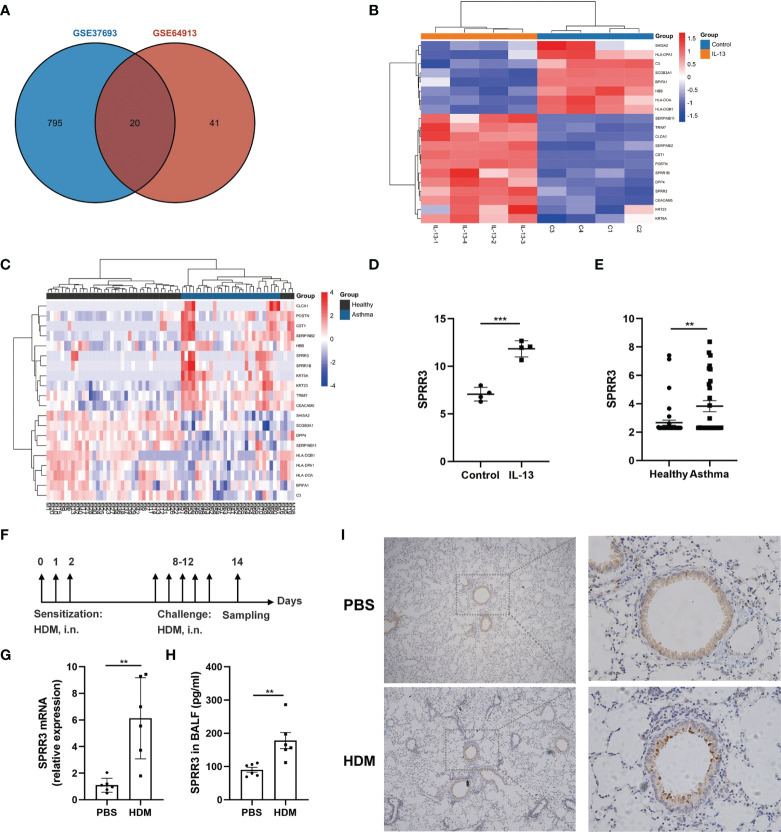
Screening and confirmation of differentially expressed genes (DEGs). **(A)** Venn diagram indicating the intersection of DEGs in datasets GSE64913 and GSE37693. **(B)** Heatmap of candidate DEGs in the GSE37693 dataset. Red represents upregulated DEGs, and blue represents downregulated DEGs. **(C)** Heatmap of candidate DEGs in the GSE64913 dataset. Red represents upregulated DEGs, and blue represents downregulated DEGs. **(D)** The expression of SPRR3 in airway epithelial cells treated with IL-13. **(E)** The expression of SPRR3 in asthmatic bronchial epithelial cells. **(F)** Schematic of the house dust mite (HDM)-induced asthmatic mouse model. **(G)** The mRNA expression of SPRR3 in lungs of HDM-induced mice. **(H)** The protein level of SPRR3 in bronchoalveolar lavage fluid (BALF) determined by ELISA. **(I)** Immunohistochemistry staining of the lung to assess the expression of SPRR3. Original magnification, ×200. Values represent mean ± SEM, n = 4–6 for each group. ^**^ *p* < 0.01, ^***^ *p* < 0.001.

Screening with a threshold of *p*-value <0.05 and |LogFC| ≥ 1 led to the identification of 815 DEGs in the IL-13-treated airway epithelial cells and 61 DEGs in the bronchial epithelial cells of asthma patients (**Figure 1A**). Twenty candidate DEGs were found in the above two datasets. The heatmap of these 20 candidate DEGs in airway epithelial cells treated with IL-13 and asthmatic bronchial epithelial cells are shown in **Figures 1B, C**. With the exception of *HBB*, *SERPINB11*, and *DPP4*, the trend of other candidate DEGs was consistent between the above two datasets. Candidate DEGs including *SERPINB2*, *POSTN*, and *CLCA1* have already been reported to participate in asthma (22). Among the remaining candidate DEGs, the expressions of two members of the SPRRs family (*SPRR1B* and *SPRR3*) were upregulated (**Figures 1B–E**). While the roles of SPRR1B and SPRR3 in asthma are unclear, it has been shown that the members of the SPRRs family SPRR2A and SPRR2B are upregulated in an IL-13-dependent manner in an allergen-induced asthma model (14). Here,we use HDM to establish an asthma mouse model (**Figure 1F**), and confirmed via quantitative PCR that the mRNA expression of SPRR3 was increased in the lung tissues of asthmatic mice (**Figure 1G**). The protein level of SPRR3 in BALF was increased in asthmatic mice (**Figure 1H**). SPRR3 protein levels were increased in the lung tissues of asthmatic mice and mainly expressed in the airway epithelium (**Figure 1I**). *In vitro*, the SPRR3 protein level was detected using airway epithelial cells treated with HDM or IL-13. SPRR3 protein levels were increased after stimulation (**Supplementary Figures 1A, B**).

### SPRR3 Knockdown Alleviates House Dust Mite-Induced Airway Inflammation and Mucus Secretion in Mice

**Figure 2:**
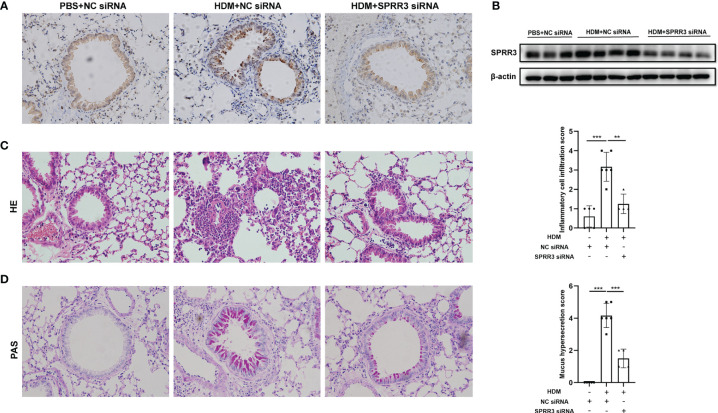
SPRR3 knockdown alleviates house dust mite (HDM)-induced airway inflammation and mucus secretion in mice. **(A)** Immunohistochemistry staining of the lung to assess the expression of SPRR3 (original magnification, ×200). **(B)** SPRR3 protein levels in mouse lungs determined by Western blotting. **(C)** Representative lung sections stained with H&E (original magnification, ×200) and histopathological scores of airway inflammations. **(D)** Representative lung sections stained with periodic acid-Schiff (PAS) (original magnification, ×200) and histopathological scores of airway mucus secretion. Values represent mean ± SEM, n = 4–6 for each group. ^**^ *p* < 0.01, ^***^ *p* < 0.001.

The asthma model was established by HDM sensitization and challenge. Airway SPRR3 expression was knocked down by transfection with siRNA against SPRR3 before the HDM challenge. SPRR3 was increased after the HDM challenge but suppressed after SPRR3 knockdown (**Figures 2A, B**). We found that the lung tissue of asthmatic mice exhibited typical inflammatory changes with increased inflammatory cell infiltration into the peribronchial and perivascular spaces. However, decreased infiltration of inflammatory cells was observed in asthmatic mice pretreated with SPRR3 siRNA (**Figure 2C**). Compared with PBS + NC siRNA group, mucus secretion was increased in the HDM + NC siRNA group, while SPRR3 knockdown significantly alleviated HDM-induced mucus overproduction (**Figure 2D**). These results indicated that SPRR3 knockdown inhibited allergic airway inflammation and mucus secretion.

### SPRR3 Knockdown Decreases the Number of Inflammatory Cells in Bronchoalveolar Lavage Fluid

**Figure 3:**
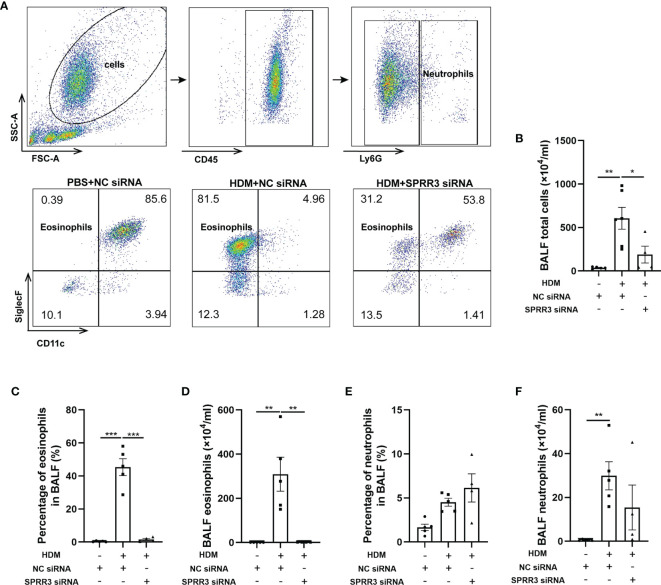
SPRR3 knockdown decreases the number of inflammatory cells in bronchoalveolar lavage fluid (BALF). Flow cytometry was used to analyze the inflammatory cells in BALF. Among BALF cells, neutrophils were gated as CD45^+^Ly6G^+^, and eosinophils were gated as CD45^+^Ly6G^−^CD11c^−^SiglecF^+^. **(A)** Flow cytometric dot plots of CD11c^−^SiglecF^+^ eosinophils gated on CD45^+^Ly6G^−^ cells. **(B)** The counts of BALF total cells. **(C)** The percentage of eosinophils in BALF. **(D)** BALF eosinophil counts. **(E)** The percentage of neutrophils in BALF. **(F)** BALF neutrophil counts. Values represent mean ± SEM, n = 4–6 for each group. ^*^ *p* < 0.05, ^**^ *p* < 0.01, ^***^ *p* < 0.001.

The inflammatory cells in BALF were tested by flow cytometry applying a gating strategy shown in **Figure 3A**. The numbers of total cells and eosinophils in BALF were markedly increased in asthmatic mice compared with those in the control group. Pretreatment with SPRR3 siRNA before the HDM challenge significantly decreased total cells and eosinophils in BALF (**Figures 3B–D**). The numbers of neutrophils were also increased significantly in BALF and then partially inhibited by SPRR3 siRNA with no statistical significance (**Figures 3E, F**).

### SPRR3 Knockdown Inhibits the Expression of IL-33 and ILC2 Accumulation in Mice

**Figure 4:**
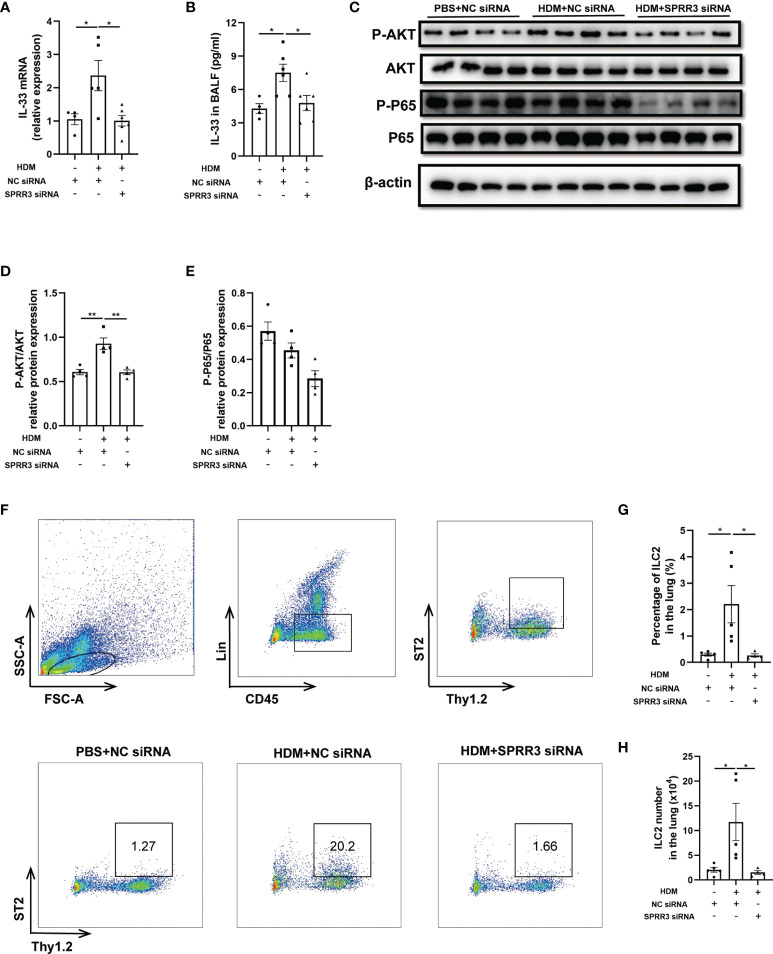
SPRR3 knockdown inhibits the expression of IL-33 and ILC2 accumulation in mice. **(A)** The mRNA expression of IL-33 measured using quantitative PCR. **(B)** The protein levels of IL-33 in bronchoalveolar lavage fluid (BALF) determined by ELISA. **(C)** Western blotting analysis of AKT and NF-κB signaling pathway activation. **(D)** Quantification of phosphorylation AKT normalized to total AKT, shown as relative expression. **(E)** Quantification of phosphorylation NF-κB P65 normalized to total NF-κB P65, shown as relative expression. **(F)** Flow cytometric dot plots of ST2^+^Thy1.2^+^ILC2s gated on CD45^+^LIN^−^ cells. **(G)** Percentage of ILC2 in the lung. **(H)** ILC2 numbers in the lung. Values represent mean ± SEM, n = 4–6 for each group. ^*^ *p* < 0.05, ^**^ *p* < 0.01.

Airway epithelial cytokines IL-25, IL-33, and TSLP play important roles in the innate immune response to allergens and promote Th2 immune responses in asthma. However, the relationship between epithelial cytokines and SPRR3 is still unknown. The HDM challenge increased IL-33 levels in the lung and BALF of mice, whereas this trend was significantly suppressed in SPRR3-knockdown mice (**Figures 4A, B**). Serving as a critical cytokine for the activation of mouse and human ILC2s, IL-33 induces rapid proliferation of ILC2s and cytokine production mainly *via* NF-κB signaling pathways. The AKT and NF-κB pathways in ILC2s were activated after treatment with IL-33, and IL-33-induced ILC2 migration was significantly blocked by specific inhibitors of the pathways (20, 23). Herein, the PI3K/AKT/NF-κB pathway was explored and found to be activated in asthmatic mice, while SPRR3 knockdown inhibited the phosphorylation of the pathway (**Figures 4C–E**).

ILC2 plays an important role in initiating and promoting Th2 inflammatory response (24). Here, lung ILC2s from mice were marked as CD45^+^Thy1.2^+^Lin^−^, and ST2-positive ILC2s were identified as a mature and activated phenotype (**Figure 4F**). The percentage of ST2-positive ILC2 cells in asthmatic mice was significantly increased compared with that in the control group. SPRR3 knockdown decreased ST2-positive ILC2 numbers (**Figures 4F–H**). Therefore, these results suggested that SPRR3 knockdown reduced the expression of IL-33 and further attenuated the activation of the PI3K/AKT/NF-κB signaling pathway in the recruitment of ILC2s to inhibit allergic airway inflammation.

### SPRR3 Contributes to the Upregulation of Alarmins IL-25, IL-33, and Thymic Stromal Lymphopoietin After Exposure to Allergen *In Vitro*

**Figure 5:**
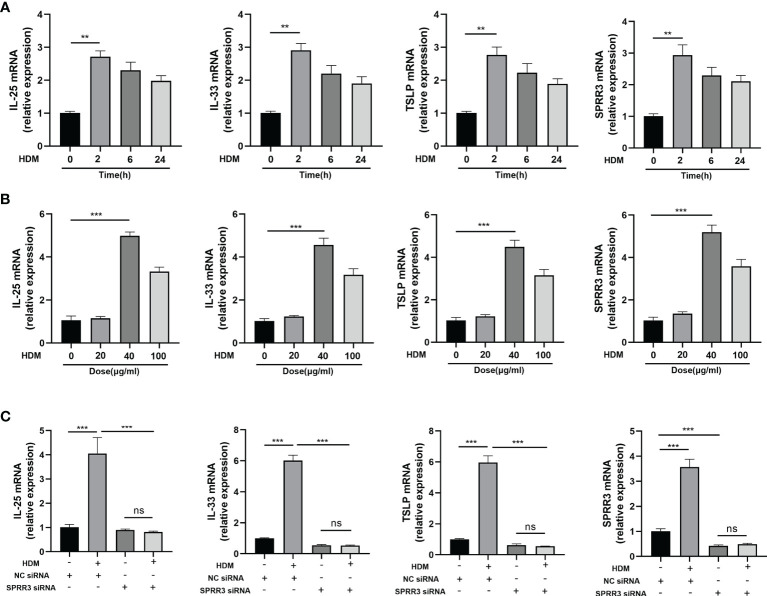
SPRR3 contributes to the upregulation of alarmins IL-25, IL-33, and thymic stromal lymphopoietin (TSLP) after exposure to allergens *in vitro*. **(A)** mRNA expressions of IL-25, IL-33, TSLP, and SPRR3 in BEAS-2B cells at different times after exposure to house dust mite (HDM) (40 μg/ml). **(B)** mRNA expressions of IL-25, IL-33, TSLP, and SPRR3 in BEAS-2B cells exposed to different concentrations of HDM. **(C)** mRNA expressions of IL-25, IL-33, TSLP, and SPRR3 in BEAS-2B cells after transfection with SPRR3 siRNA or negative control siRNA prior to HDM exposure. Values represent mean ± SEM, n = 3 independent experiments. ^**^ *p* < 0.01, ^***^ *p* < 0.001. ns, no significant difference.

We next sought to establish an *in vitro* system to confirm and extend our *in vivo* finding that airway SPRR3 plays a key role in IL-25, IL-33, and TSLP expression after HDM exposure. BEAS-2B cells were stimulated with HDM to simulate airway epithelial allergen exposure. The mRNA expressions of IL-25, IL-33, and TSLP in BEAS-2B cells were the highest after stimulation with HDM for 2 h compared with those in the control groups, and the trend of SPRR3 expression was consistent with the increased epithelial cytokine production (**Figure 5A**). When stimulated with HDM at 40 μg/ml, SPRR3, IL-25, IL-33, and TSLP exhibited the greatest increase (**Figure 5B**). After transfection, SPRR3 knockdown significantly suppressed the expression of IL-25, IL-33, and TSLP (**Figure 5C**). These data indicated that SPRR3 was involved in allergen-induced IL-25, IL-33, and TSLP expression in human airway epithelial cells.

### SPRR3 Is Involved in Th2 Immune Responses in the House Dust Mite-Induced Asthma Model

**Figure 6:**
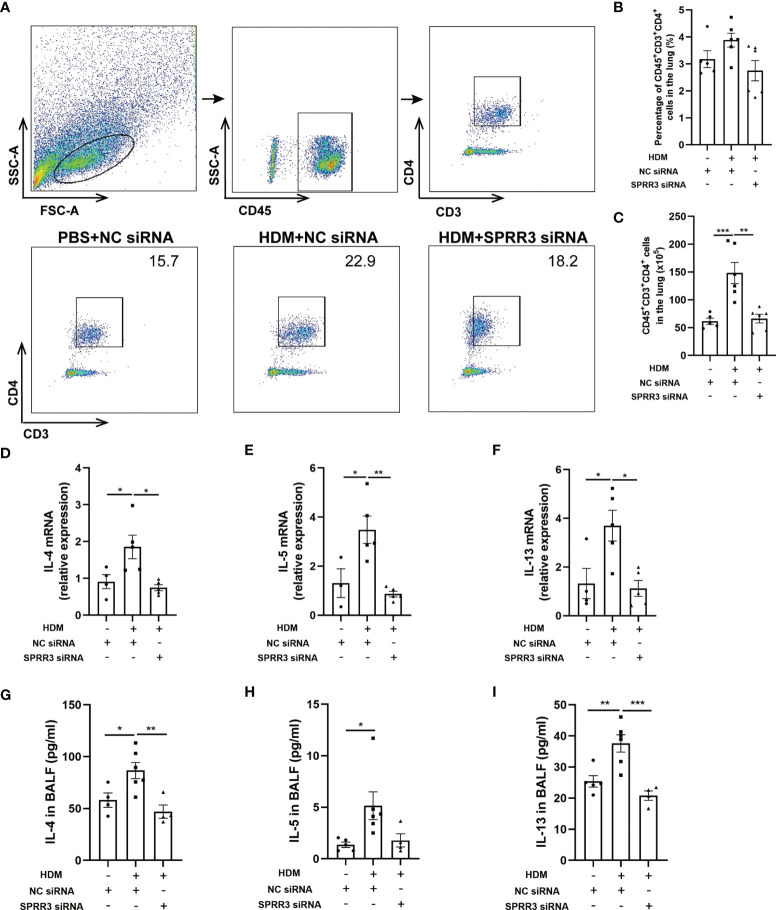
SPRR3 is involved in Th2 immune responses in the house dust mite (HDM)-induced asthma model. **(A)** Gating strategy to identify CD4^+^ T cells in mouse lungs, cells can be separated in CD45^+^ and CD45^−^ cell populations. Gating for CD4^+^ T cells (CD3^+^CD4^+^) within the CD45^+^ cell population. **(B)** The percentage of CD4^+^ T cells in the lung. **(C)** The counts of CD4^+^ T cells in the lung. **(D–F)** The mRNA expressions of IL-4, IL-5, and IL-13 measured using quantitative PCR. **(G–I)** The protein levels of IL-4, IL-5, and IL-13 in bronchoalveolar lavage fluid (BALF) determined by ELISA. Values represent mean ± SEM, n = 4–6 for each group. ^*^ *p* < 0.05, ^**^ *p* < 0.01, ^***^ *p* < 0.001.

Studies have shown that ILC2s potentiate CD4 T helper cell activation in asthma, which is the effector cell in the complex pathophysiology of inflammation in asthma (25, 26). We further explored the influence of SPRR3 on CD4^+^ T cells in SPRR3-knockdown mice. Lung tissues were prepared as a single-cell suspension, and the number of CD4^+^ T cells in lung tissue was measured by flow cytometry (**Figure 6A**). The numbers of CD4^+^ T cells were increased in mice challenged with HDM. However, after HDM exposure, SPRR3-knockdown mice had diminished numbers of CD4^+^ T cells (**Figures 6B, C**).

We further analyzed the expression of Th2 cytokines IL-4, IL-5, and IL-13 in the lungs of mice by quantitative PCR. Lung IL-4, IL-5, and IL-13 mRNA levels were upregulated after HDM sensitization and challenge. However, the trend of increasing levels of these cytokines was significantly suppressed after SPRR3 knockdown (**Figures 6D–F**). The levels of IL-4, IL-5, and IL-13 in BALF were increased in asthmatic mice but decreased after SPRR3 knockdown, consistent with changes in mRNA levels in lung tissue (**Figures 6G–I**).

## Discussion

In this study, bioinformatics analysis of GEO databases revealed that *SPRR3* gene expression was significantly increased in asthma. We further confirmed that SPRR3 expression was markedly increased in lung tissues exposed to HDM. Airway inflammation, accompanied by infiltrated eosinophils, enhanced Th2 responses, and ILC2 accumulation, was also observed in the HDM-induced asthmatic mice, which was then abrogated by knockdown of SPRR3. Moreover, SPRR3 inhibition suppressed the expression of IL-33 in lungs and BALF of asthmatic mice and further inhibited the activation of the PI3K/AKT/NF-κB pathway in the HDM-induced lung. Of note, SPRR3 inhibition also alleviated HDM-induced inflammatory responses in BEAS-2B cells by decreasing IL-25, IL-33, and TSLP expression. These findings are the first to illustrate the effects of SPRR3 on allergic airway inflammation.

Previous studies found that SPRRs play key roles in allergic asthma. SPRR2A and SPRR2B were found to be increased in ovalbumin and an *Aspergillus fumigatus*-induced asthma model. *In situ* hybridization revealed that SPRR2 was mainly distributed in bronchial epithelial cells and mononuclear cells associated with inflammation after the allergen challenge. Notably, the Th2 cytokine IL-13 potently induced SPRR2 expression in the lung, suggesting that pulmonary SPRR2 expression was largely IL-13 and STAT6-dependent (14). Interestingly, SPRR3, another SPRR member mapped to the same chromosome region of SPRR2 (9), was found to be increased in lung tissue exposed to HDM (especially in the airway epithelium) in our study.

The airway epithelium is usually the first line of defense that is exposed to inhaled allergens and pathogens, releasing alarmins (including IL-25, IL-33, and TSLP) to propagate the development and exacerbation of asthma (27, 28). The alarmins, which are secreted by the airway epithelium, alert the immune system to external insults and regulate tissue restoration and repair after injury (8, 29). ILCs are increasingly recognized as a key component of innate immunity; among these, ILC2s are particularly abundant in the skin, lung, and adipose tissue. ILC2s are activated by epithelial cytokines such as IL-25 and IL-33 and produce large amounts of IL-5 and IL-13, which are required for the initiation of type 2 immune responses (20, 30–32). Many studies have shown that ILC2s were increased in the blood and sputum in patients with asthma, especially in those with type 2 high severe eosinophilic asthma (33–35). Besides, IL-25, IL-33, and TSLP can directly program dendritic cells to promote the activation of CD4^+^ T cells or indirectly mediated by ILC2s (5, 36). The major pathways involved in airway inflammation (IL-4, IL-13, and IL-5 signaling) can be driven by CD4^+^ T cells (5). The depletion of CD4^+^ T cells in asthmatic mice prevented airway inflammation (37). Despite that ILC2s and CD4^+^ T helper 2 (Th2) cells have overlaps in their functions, ILC2s, as one of the early and critical signals, are commonly placed at the center of Th2 immune responses (32). In our study, knockdown of SPRR3 suppressed the expression of IL-33 in the lung and BALF and inhibited lung ILC2 accumulation in the asthmatic mice. Decreased IL-25, IL-33, and TSLP expressions were observed in bronchial epithelial cells exposed to allergens after SPRR3 siRNA treatment. SPRR3 knockdown also inhibited the induction of CD4^+^ T cells in the lung tissues and reduced Th2 cytokine production. This indicates that SPRR3 acts as a key molecule in airway eosinophilic inflammation by regulating alarmin release, ILC2 accumulation, and Th2 immune response.

IL-33 participates in the activation of NF-κB signaling and also induces the phosphorylation and activation of PI3K/AKT signaling modules, resulting in the production and release of pro-inflammatory cytokines (38). In asthma, IL-33 is defined as an “activating cytokine” that induces proliferation and type 2 cytokine production by ILC2s *via* the NF-κB signaling pathway (23). Moreover, IL-33-induced ILC2 migration involves the activation of AKT and NF-κB pathways. Flow cytometric analysis revealed that IL-33 markedly increased the phosphorylation of AKT and NF-κB in ILC2. Inhibiting these signaling pathways significantly suppressed IL-33-induced ILC2 migration (20). SPRR3 was reported to promote phosphorylation of AKT in tumors such as breast cancer, colorectal tumor, and lung cancer (12, 39, 40). Our study confirmed that the PI3K/AKT/NF-κB pathway was activated in the HDM-induced mouse model and further abrogated by knockdown of SPRR3, consistent with previous studies in tumors (12). These results suggest that SPRR3 knockdown reduces the expression of IL-33 and further attenuates ILC2 recruitment through the PI3K/AKT/NF-κB signaling pathway to inhibit allergic airway inflammation.

Numerous studies have demonstrated that the PI3K/AKT/NF-κB pathway participates in the occurrence of asthma and is related to allergic airway inflammation and Th cell differentiation, especially airway remodeling (41–44). Lin et al. found that enhanced PI3K/AKT/NF-κB signaling occurred in mast cell activation, which further promoted allergic airway inflammation (45). In ovalbumin-induced asthmatic mice, the inactivation of the PI3K/AKT/NF-κB pathway suppressed airway smooth muscle cell proliferation and airway remodeling (46). Our study suggests that SPRR3 knockdown may attenuate recruitment of ILC2 through the PI3K/AKT/NF-κB signaling pathway. Whether SPRR3 regulates other pathological processes of asthma through the PI3K/AKT/NF-κB signaling pathway remains unclear. Further studies are required to unravel the role of SPRR3 in Th cell differentiation and airway remodeling.

Our study has several limitations. First, although the lineage antibody cocktail (including five lineage antibodies) we used for the depletion of major hematopoietic cells to identify ILC2s has been evidenced previously, the gating strategy of ILC2 could be further optimized by adding additional markers as reported (32, 47). Second, all of the *in vivo* data were based on the knockdown of SPRR3 expression in the lungs of mice, but not in SPRR3-knockout mice. Therefore, further work on SPRR3-knockout mice is essential to validate the findings of the current study and clarify the mechanism of the SPRR3-mediated PI3K/AKT/NF-κB pathway in more detail in asthma.

In summary, we found that SPRR3 played key roles in HDM-induced allergic airway inflammation by regulating the IL-33/ILC2 axis and Th2 cytokine release. Inhibition of upregulated SPRR3 reduced the expression of IL-33 and further attenuated the activation of the PI3K/AKT/NF-κB signaling pathway in the recruitment of ILC2s to inhibit allergic airway inflammation. *In vitro*, SPRR3 knockdown also inhibited IL-25, IL-33, and TSLP expression in human airway epithelial cells upon HDM exposure. Our study shows that SPRR3 acts as a key upstream modulator in airway epithelial inflammation and Th2 responses, potentially serving as a novel therapeutic target for the treatment of allergic asthma.

## Data Availability Statement

The raw data supporting the conclusions of this article will be made available by the authors, without undue reservation.

## Ethics Statement

The animal study was reviewed and approved by the Animal Care and Use Committee of Zhongshan Hospital, Fudan University.

## Author Contributions

GZ, HC, JW, and MJ designed the study. GZ, HC, XS, and XG analyzed the data, prepared the figures, and drafted the manuscript. HC, LY, YM, MZ, YZ, and CY interpreted the data and revised the manuscript. JW and MJ provided overall supervision and revised the manuscript. All authors contributed to the article and approved the submitted version.

## Funding

This study was sponsored by the National Natural Science Foundation of China (8197010253, 82000013), the Clinical Research Project of Zhongshan Hospital (2020ZSLC26), Shanghai Sailing Program (20YF1405700), and the National Key R&D Program of China (2017YFC0910003 and 2017YFC0910000).

## Conflict of Interest

The authors declare that the research was conducted in the absence of any commercial or financial relationships that could be construed as a potential conflict of interest.

## Publisher’s Note

All claims expressed in this article are solely those of the authors and do not necessarily represent those of their affiliated organizations, or those of the publisher, the editors and the reviewers. Any product that may be evaluated in this article, or claim that may be made by its manufacturer, is not guaranteed or endorsed by the publisher.
