# Supplementary material for: Small Proline-Rich Protein 3 Regulates IL-33/ILC2 Axis to Promote Allergic Airway Inflammation
Source: Front Immunol. 2022 Jan 20;12:758829. doi: 10.3389/fimmu.2021.758829 (PMC8810634; doi:10.3389/fimmu.2021.758829)

**Supplementary Figure 1:** **SPRR3 protein level was increased after stimulation with HDM or IL-13. (A)** Western blot analysis of SPRR3 in airway epithelial cells treated with HDM(40μg/ml). **(B)** Western blot analysis of SPRR3 in airway epithelial cells treated with IL-13(20ng/ml).


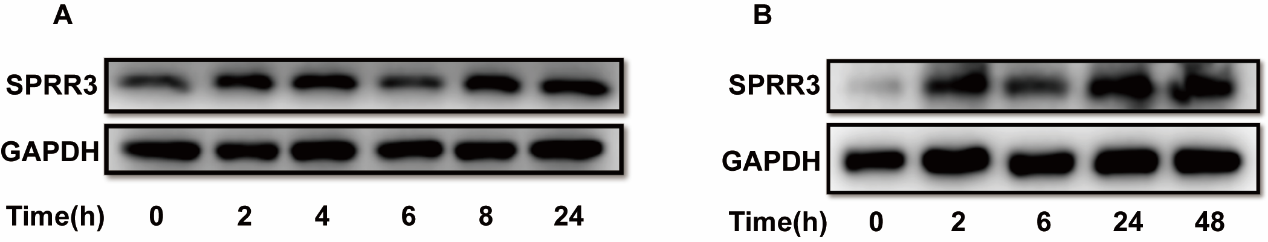

Supplement: Supplementary file 1 [file DataSheet_1.docx]
